# Effects of Glucagon-Like Peptide-1 Receptor Agonists on Weight Loss in Patients with Type 2 Diabetes: A Systematic Review and Network Meta-Analysis

**DOI:** 10.1155/2015/157201

**Published:** 2015-01-20

**Authors:** Feng Sun, Sanbao Chai, Lishi Li, Kai Yu, Zhirong Yang, Shanshan Wu, Yuan Zhang, Linong Ji, Siyan Zhan

**Affiliations:** ^1^Department of Epidemiology and Biostatistics, School of Public Health, Peking University Health Science Centre, 38 Xueyuan Road, Haidian District, Beijing 100191, China; ^2^Department of Preventive Medicine, College of Medicine, Shihezi University, Shihezi 832002, China; ^3^Department of Physiology, Capital Medical University, Beijing 100069, China; ^4^Department of Statistics, Graduate School of Arts and Sciences, Columbia University, New York, NY 10027, USA; ^5^Department of Orthopedics, Tianjin Fifth Central Hospital, Tianjin 300450, China; ^6^Shantou-Oxford Clinical Research Unit, Shantou University Medical College, Shantou 515041, China; ^7^Department of Endocrinology and Metabolism, People's Hospital, Peking University, Beijing 100044, China

## Abstract

To evaluate the effectiveness of glucagon-like peptide-1 receptor agonists (GLP-1 RAs) on weight reduction in patients with Type 2 diabetes mellitus (Type 2 DM), a network meta-analysis was conducted. MEDLINE, EMBASE, Cochrane Library, and ClinicalTrials.gov were searched from 1950 to October 2013. Randomized controlled trials (RCTs) involving GLP-1 RAs were included if they provided information on body weight. A total of 51 RCTs were included and 17521 participants were enrolled. The mean duration of 51 RCTs was 31 weeks. Exenatide 10 *μ*g twice daily (EX10BID) reduced weight compared with exenatide 5 *μ*g twice daily (EX5BID), liraglutide 0.6 mg once daily (LIR0.6QD), liraglutide—1.2 mg once daily (LIR1.2QD), and placebo treatment, with mean differences of −1.07 kg (95% CI: −2.41, −0.02), −2.38 kg (95% CI: −3.71, −1.06), −1.62 kg (95% CI: −2.79, −0.43), and −1.92 kg (95% CI: −2.61, −1.24), respectively. Reductions of weight treated with liraglutide—1.8 mg once daily (LIR1.8QD) reach statistical significance (−1.43 kg (95% CI: −2.73, −0.15)) versus LIR1.2QD and (−0.98 kg (95% CI: −1.94, −0.02)) versus placebo. Network meta-analysis found that EX10BID, LIR1.8QD, and EX2QW obtained a higher proportion of patients with weight loss than other traditional hypoglycemic agents. Our results suggest GLP-1 RAs are promising candidates for weight control in comparison with traditional hypoglycemic drugs, and EX10BID, LIR1.8QD, and EX2QW rank the top three drugs.

## 1. Introduction

Glucagon-like peptide-1 (GLP-1) is a gut hormone, secreted from the intestine in response to meal ingestion, which stimulates insulin secretion and inhibits glucagon release in a dose-dependent fashion [1]. GLP-1 can suppress food intake and appetite and decelerate gastric emptying and induce satiety, so it plays an important role in regulation of blood glucose [2, 3]. But the rapid inactivation of GLP-1 in vivo and the consequent short half-life prevents its therapeutic use. Long acting GLP-1 receptor agonists (GLP-1 RAs) that can be administered via subcutaneous injection once or twice a day or even once a week have been developed [4]. GLP-1 RAs include exenatide, liraglutide, albiglutide, taspoglutide, lixisenatide and LY2189265. Treatment with GLP-1 RAs improves insulin resistance and glucose homoeostasis for patients with Type 2 DM [5, 6]. Also they have a rather low risk of hypoglycemia because of their mode of action. Exenatide and liraglutide are currently successfully being employed in the treatment of Type 2 DM [7].

Overweight or obesity may increase the risk of cardiovascular complications as well as resulting in serious psychological distress for most patients with Type 2 DM [8]. Effective interventions designed to achieve weight reduction are a critical part of Type 2 DM management to prevent the development of microvascular and macrovascular complications. But many antidiabetic agents (insulin, thiazolidinediones, and sulfonylureas) have side-effect of bodyweight increase. GLP-1 can stimulate satiety by binding to its receptor on hypothalamic neurons and reduce calorie ingestion by delayed gastric emptying [9, 10]. Although several meta-analyses have revealed the association between GLP-1 RAs and weight loss, all of them are head-to-head comparisons of GLP-1 RAs versus placebo or active comparator drugs [7, 11–13], and these analyses did not evaluate the impact of different doses of GLP-1 RAs on weight loss. Therefore, we did network meta-analysis of GLP-1 RAs in order to provide up-to-date overview of the effects of GLP-1 RAs on weight control in patients with Type 2 DM.

## 2. Materials and Methods 

### 2.1. Search Strategy

In consultation with a medical librarian, we established a search strategy for the MEDLINE, EMBASE, and Cochrane Library (from 1950 to October 2013). The following search strategy (Ovid) was adapted for the other databases: (1) exp glucagon-like peptide-1 agonists/; (2) (glucagon-like peptide^*^ or GLP-1).tw.; (3) (exenatide or liraglutide).tw.; (4) randomized controlled trial.pt.; (5) (randomized or randomised).tw.; (6) ((1) or (2) or (3)) and ((4) or (5)). We also searched ClinicalTrials.gov for (unpublished) completed trials. In addition, we searched the bibliographies of published systematic reviews [7, 11–13]. All relevant authors and principal investigators were contacted to supplement incomplete reports of the original papers or to provide data for unpublished studies.

### 2.2. Inclusion and Exclusion Criteria

All the studies included are in English and they are eligible for inclusion only if they were RCTs involving GLP-1 RAs, other hypoglycemic drugs or placebo with complete data on body weight. Trials are excluded if only they meet one of the following: (1) trials are not RCT (e.g., review, expert comment, editor opinion, new agent introduction, single case report, or case series); (2) early studies; (3) experimentation on animals or in vitro; (4) not conducted in Type 2DM; (5) pharmacokinetics research; (6) trials underway, unfinished, or suspended; (7) economical evaluation research; (8) other unrelated researches. These studies were approved by the local ethics committees and written informed consent was obtained from all the patients.

### 2.3. Clinical Doses of GLP-1 RAs

We only included doses that are likely to be used in routine clinical care. We excluded trials or arms using nonstandard doses, which mainly came from dose-ranging studies. So only those dose-arms possibly relevant to clinical application were included in our study. The standard exenatide regimens are 5 *μ*g twice daily (EX5BID), 10 *μ*g twice daily (EX10BID), and 2 mg once weekly (EX2QW). The standard liraglutide regimens are 0.6 mg once daily (LIR0.6QD), 0.9 mg once daily (LIR0.9QD), 1.2 mg once daily (LIR1.2QD), and 1.8 mg once daily (LIR1.8QD), respectively.

### 2.4. Data Extraction and Quality Evaluation

After obtaining full reports of the candidate trials, two reviewers (CSB and LLS) independently used ADDIS software [14] to extract information concerning population characteristics (age, T2D course, baseline HbA_1c_) and weight change of each treatment group. Quality of studies was assessed according to JADAD scale [15]: adequate method for randomization, appropriate blinding procedures, and detailed report of withdrawals. We resolved differences in extraction through discussion and consensus.

### 2.5. Data Analysis

#### 2.5.1. Methods for Direct Treatment Comparisons

Initially, we performed standard pairwise meta-analyses using the DerSimonian-Laird random effects model [16] for every treatment comparison. Where studies did not report intent-to-treat, we analyzed outcomes as all-patients randomized. The *I* ^2^ statistic was calculated as a measure of the proportion of the overall variation that is attributable to between-study heterogeneity [17].

#### 2.5.2. Network Meta-Analysis

We did a network meta-analysis within a Bayesian framework [18], and we summarized the results using mean difference (MD) and credible interval (CrI). Bayesian network meta-analysis was a generalization of traditional meta-analysis that allowed all evidence to be taken into account simultaneously (both direct and indirect). We estimated the posterior densities for all unknown parameters using MCMC (Markov chain Monte Carlo) for each model. Each chain used 40,000 iterations with a burn-in of 20,000. To formally check whether a model's overall fit was satisfactory, we considered an absolute measure of fit: ${\overset{-}{D}}_{\text{res}}$. This is the posterior mean of the deviance under the current model, minus the deviance for the saturated model. We would expect that each data point should contribute about 1 to the posterior mean deviance so that it can be compared with the number of data points for the purpose of checking model fit [19]. We estimated the ranking probabilities for all treatments at each possible rank for every intervention. Then, we obtained a treatment hierarchy using the surface under the cumulative ranking curve (SUCRA) [20] and posterior mean ranks. SUCRA can also be expressed as a percentage interpreted as the percentage of efficacy of a treatment on weight control that would be ranked first without uncertainty.

#### 2.5.3. Assessment of Statistical Inconsistency

One key assumption of the network meta-analysis models is the consistency between direct and indirect evidence, that is, if the information of both sources of evidence is similar enough in order to be combined. To evaluate the presence of inconsistency locally we will use the loop-specific approach [21]. This method evaluates the consistency assumption in each closed loop of the network separately as the difference between direct and indirect estimates for a specific comparison in the loop (inconsistency factor). Then, the magnitude of the inconsistency factors and their 95% CIs can be used to infer the presence of inconsistency in each loop. Inconsistency can be evaluated as the disagreement between different sources of evidence within a closed loop [22].

Analyses were conducted using STATA 11.0 (pairwise meta-analysis, *I* ^2^ calculations, and estimation of inconsistence), R 3.0.2 (SUCRA graphs) and WinBUGS 1.4.3 (network meta-analysis and model fit).

## 3. Results

### 3.1. Literature Search Results and Study Characteristics

**Figure 1:**
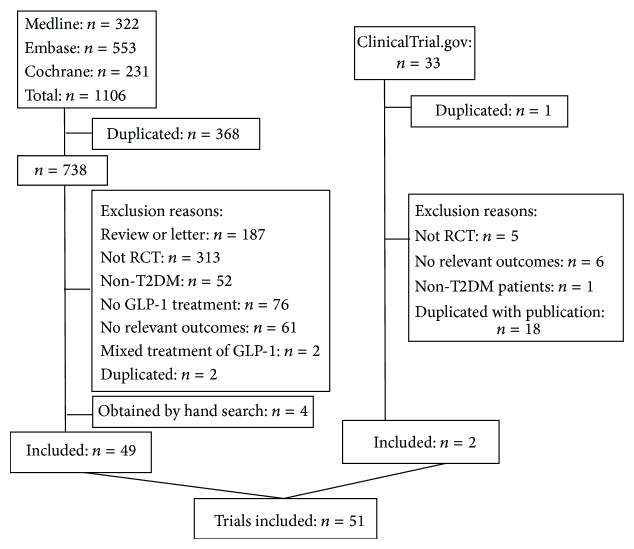
Flowchart of inclusion and exclusion.

1139 studies were identified at first. Finally, 51 studies involving 13 treatments and 17521 participants with a mean duration of 31 weeks were included. Flowchart of inclusion and exclusion was showed in Figure 1.

#### 3.1.1. Study Characteristics

Table 1 summarized the characteristics of the included 51 studies. Publication year varied from 2002 to 2013 and duration ranged from 4 weeks to 234 weeks. The 17521 patients had a mean baseline HbA_1c_ of 8.3%, a mean age of 56 y, and a mean diabetes duration of 8.3 y. There were 36 studies about exenatide, 13 studies about liraglutide, and 2 studies for both [23, 24].

#### 3.1.2. Evidence Network

**Figure 2:**
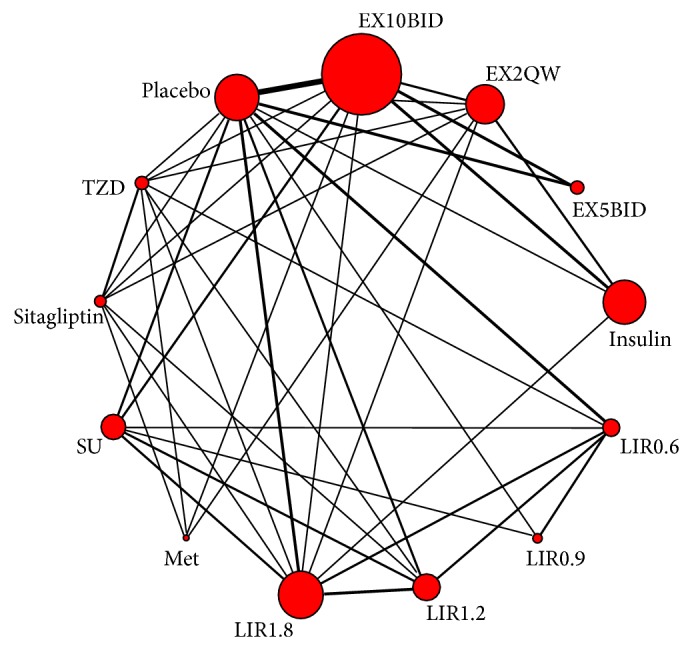
Network of clinical trials about GLP-1 RAs and other hypoglycemic drugs or placebo in patients with Type 2 diabetes. Lines connect the interventions that have been studied in head-to-head (direct) comparisons in the eligible RCTs. The width of the lines represents the cumulative number of RCTs for each pairwise comparison and the size of every node is proportional to the number of randomized participants (sample size). EX5BID: exenatide 5 *μ*g twice daily; EX10BID: exenatide 10 *μ*g twice daily; EX2QW: exenatide 2 mg once weekly; LIR0.6: liraglutide 0.6 mg once daily; LIR0.9: liraglutide 0.9 mg once daily; LIR1.2: liraglutide 1.2 mg once daily; LIR1.8: liraglutide 1.8 mg once daily; SU: sulfonylureas; TZD: thiazolidinediones; Met: metformin.

Thirteen treatments were analyzed, including seven GLP-1 RAs dose-group (EX10BID, EX2QW, EX5BID, LIR0.6QD, LIR0.9QD, LIR1.2QD, and LIR1.8QD), five kinds of traditional antidiabetics (insulin, metformin (Met), sulfonylureas (SU), sitagliptin, and thiazolidinediones (TZD)), and placebo. Thirty-two trials (62.75%) were two-arm studies and nineteen (37.25%) were multiple-arm studies (see Table 1 and Figure 2), totally 127 arms. The maximum studies about EX10BID were fourteen.

#### 3.1.3. Methodological Quality of Studies

We found that the reporting quality of studies varied. The overall quality of studies was rated according to JADAD scale. The proportion of appropriate description of randomization, allocation concealment, blinding, and dropout were 78.43% (40/51), 50.98% (26/51), 52.94% (27/51), and 88.24% (45/51), respectively. Additionally, 90.20% (46/51) trials were used intention-to-treat analysis (see Table 1 in Supplementary Material available online at http://dx.doi.org/10.1155/2015/157201). Overall risk of bias was, respectively, low and bias mainly came from allocation concealment and blinding of outcome assessment.

### 3.2. Direct Pairwise Meta-Analysis and Network Meta-Analysis

#### 3.2.1. Direct Pairwise Meta-Analysis about the Impact of GLP-1 RAs on Weight Control

Table 2 shows the outcome of direct meta-analysis. Compared with placebo, EX10BID and LIR1.8QD decreased body weight by −1.38 kg (95% CI: −1.74, −1.03) and −1.32 kg (95% CI: −2.22, −0.43), respectively, while there was a statistically significant weight gain observed in the traditional hypoglycemic drugs (insulin, SU, and TZD) compared with placebo, with mean differences of 2.02 kg (95% CI: 1.02, 3.02), 2.03 kg (95% CI: 0.91, 3.15), and 2.20 kg (95% CI: 1.54, 2.86), respectively.

GLP-1 RAs achieved a greater weight loss than traditional hypoglycemic drugs; the range of weight reduction was −7.30 kg (95% CI: −12.82, −1.78)~−1.38 kg (95% CI: −1.74, −1.03) with exenatide and −3.12 kg (95% CI: −3.80, −2.44)~−1.40 kg (95% CI: −1.95, −0.85) with liraglutide, respectively.

#### 3.2.2. Network Meta-Analysis about the Impact of GLP-1 RAs on Weight Control

Table 2 displays the result of network meta-analysis between GLP-1 RAs and traditional hypoglycemic drugs. As displayed in the preceding pairwise meta-analysis, we found EX10BID and LIR1.8QD were associated with a significant reduction in body weight versus placebo, with mean differences of −1.92 kg (95% CI: −2.61, −1.24) and −0.98 kg (95% CI: −1.94, −0.02), respectively, while treatment with insulin, SU, and TZD resulted in significant weight gain versus placebo (range from 2.60 kg (95% CI: 1.56, 3.62) to 3.37 kg (95% CI: 2.29, 4.48)).

Similar to direct comparisons, exenatide and liraglutide showed more advantage on weight control than traditional hypoglycemic drugs. The weight reduction significantly ranged from −5.30 kg (95% CI: −6.38, −4.24) to −2.21 kg (95% CI: −3.59, −0.83) with exenatide and from −4.35 kg (95% CI: −5.45, −3.24) to −2.21 kg (95% CI: −3.92, −0.53) with liraglutide.

Compared with EX5BID, LIR0.6QD, LIR1.2QD, and placebo treatment, treatment with EX10BID resulted in a significantly greater decrease in body weight, with mean differences of −1.07 kg (95% CI: −2.41, −0.02), −2.38 kg (95% CI: −3.71, −1.06), −1.62 kg (95% CI: −2.79, −0.43), and −1.92 kg (95% CI: −2.61, −1.24), respectively. Reductions in body weight treated with LIR1.8QD reach statistical significance (−1.43 kg (95% CI: −2.73, −0.15)) versus LIR0.6QD and (−0.98 kg (95% CI: −1.94, −0.02)) versus placebo.

### 3.3. Ranking of Different Doses of GLP-1 RAs on Weight Control

Network meta-analyses enable estimation of the probability that each intervention is the best for each outcome. Probabilities for each treatment can be plotted in absolute rankograms or cumulative rankograms. A simple numerical summary supplying the graphical display of cumulative ranking is used to estimate the surface under the cumulative line for each treatment. Larger SUCRA means the drug has lower ranking (see Supplementary Figures 1(a) and 1(b)).

Table 3 showed the mean SUCRA values providing the hierarchy of 13 treatments on weight loss: EX10BID (98.30%), LIR1.8QD (78.89%), EX2QW (76.23%), EX5BID (75.02%), Met (63.32%), LIR1.2QD (58.44%), placebo (48.50%), LIR0.9QD (42.90%), sitagliptin (42.47%), LIR0.6QD (38.83%), insulin (12.86%), TZD (11.29%), and SU (3.02%), respectively. According to SUCRA values, EX10BID had the highest impact and SU had the least impact on weight loss among 13 treatments.

### 3.4. Model Fit and Inconsistency Check

The model fit can be evaluated using the posterior mean of the residual deviance ${\overset{-}{D}}_{\text{res}}$. The value of the ${\overset{-}{D}}_{\text{res}}$ was 115.41, close to 127 of the data points for weight control, meaning model's overall fit was relatively satisfactory. Additionally, statistical inconsistency between direct and indirect comparisons was generally low for weight control. Most loops (90.70%, 39/43) of 27 triangular loops and 16 quadratic loops were consistent (*P* value > 0.05; see Supplementary Table 2) and their 95% CIs included 0 according to the forest plots; that means the direct estimate of the summary effect was not different from the indirect estimate (see Supplementary Figure 2).

## 4. Discussion

Obesity is known to increase the risk of diabetes and complications such as insulin resistance, dyslipidemia, and cardiovascular diseases [25, 26]. Over 80% of individuals with Type 2 DM are overweight or obese [27]. It is attractive to both doctors and patients to avoid weight gain of Type 2 DM during the treatment of glycaemic control. GLP-1 RAs are a novel class of glucose-lowering drugs which has been shown to improve glycaemic control and promote weight loss in clinical studies of patients with Type 2 DM. In 2005, the US Food and Drug Administration approved the first long acting stable GLP-1 RA. Exenatide (Byetta; EliLilly) and liraglutide (Victoza; NovoNordisk) are now available on the market. Both drugs can be used in combination with oral antidiabetic drugs such as metformin, thiazolidinediones, or sulfonylureas compounds. The treatments are approved for patients with Type 2 DM who have not achieved adequate glycemic control after treatment with traditional antidiabetic drugs.

The network meta-analysis combines direct and indirect evidences in a single analysis, enabling simultaneously comparison of multiple interventions, which is different from the traditional meta-analysis. This approach makes use of direct comparisons from existing trials comparing 2 treatment strategies and indirect comparisons constructed from 2 trials that have at least 1 treatment in common [18]. This statistical tool preserves the within-trial, randomized comparison of each study. And Bayesian posterior probabilities were used to classify the effect of GLP-1 RAs.

Our study showed that a higher proportion of subjects experienced weight loss with exenatide (EX10BID, EX2QW, and EX5BID) and liraglutide (LIR1.2QD, LIR0.6QD, and LIR1.8QD) than with insulin, SU, and TZD, which were similar to previous meta-analysis [7, 11–13, 28], with weight loss following GLP-1 RAs treatment ranging from −3.31 to −1.22 kg. Our results indicated that subjects experienced greater weight reductions in a higher exenatide dose (EX10BID) and liraglutide dose (LIR1.8QD) compared with insulin, SU, TZD, and placebo, suggesting a dose-dependent effect. In accordance with the previous report liraglutide 1.8 mg treated subjects experienced more weight loss than 1.2 mg treated subjects [29]. Compared with placebo, treatment with SU, TZDs, and insulin resulted in a significantly greater increase in body weight, with change from 2.60 kg to 3.37 kg. By contrast, use of EX10BID and LIR1.8QD resulted in a significant decrease in bodyweight, with mean changes of −1.92 kg and −0.98 kg, respectively. These results were consistent with the previous studies [30–33]. Although the precise mechanisms associated with weight loss have not been elucidated yet, it was believed that gastric emptying was an important factor for weight loss. However, the preclinical study showed that liraglutide's effect on gastric emptying diminished over time, whereas the effect on body weight was sustained over the treatment period. Recently, evidence indicated that GLP-1-induced weight reduction required higher GLP-1 RAs levels [34].

Compared with placebo, weight did not decrease substantially either with EX2QW and EX5BID or with LIR0.6QD, LIR0.9QD, and LIR1.2QD. But the results suggested the potential relationship between weight reduction and drug dose. Except for EX10BID, body weight loss was not significant for sitagliptin and GLP-1 RAs in our meta-analysis. Like the report that showed subjects taking metformin alone lost more weight than subjects taking an SU plus metformin [35], our study showed that body weight did not change in patients with metformin compared with those with exenatide and liraglutide.

There were statistically and clinically significant differences between drug classes in weight changes. In our study, EX10BID, LIR1.8QD, and EX2QW were ranked the top three drugs in terms of weight reduction. The results suggested weight reduction of patients has a close relationship with dose of GLP-1 RAs.

There are several strengths in our analysis. First, our study is the largest evaluation of GLP-1 RAs on weight control to date. Second, the network meta-analysis technique allows dissection of the individual drug to evaluate weight control, especially facing very few RCTs which directly compared GLP-1 RAs in Type 2 DM. We applied a Bayesian model to explore the effect of indirect comparison between them, and it is thought to be the most appropriate method for multiple-treatments network meta-analysis [18]. Additionally, posterior probability from Bayesian model can help to apply the rank of GLP-1 RAs in practice.

Several limitations need to be cautious. First, other unpublished literatures on relevant pharmaceutical websites were not searched and only trials in English were included, which may lead to a potential publication bias. Second, most trials included in this review were not specially designed to evaluate body weight, with the risk of misdiagnosis and under diagnosis. Finally, lack of information about the distribution of clinical and methodological variables may lead to potential sources of either heterogeneity or inconsistency in every comparison-specific group of trials.

To sum up, this network meta-analysis provides a useful and complete picture of the associations between GLP-1 RAs, conventional antidiabetic drugs, and placebo on weight control. GLP-1 RAs are associated with significant weight loss for the treatment of Type 2 DM. However, the application of our results should take into account limitations of the analysis and the specific clinical situation. More long-term large-scale trials will be necessary to confirm potential efficacy on weight loss. Meantime, determining the specific subject-related factors associated with greater weight loss with GLP-1 receptor agonists may be helpful to clinicians in identifying patients who would likely benefit from their use.

## 5. Conclusions

GLP-1 receptor agonists cause weight reduction, in contrast to insulin, metformin, sulfonylureas, sitagliptin, thiazolidinediones, and placebo. According to the mean SUCRA values, EX10BID, LIR1.8QD, and EX2QW rank the top three drugs.

## Supplementary Material

Supplementary Table 1: The overall quality of total 51 studies was rated according to JADAD scale. The proportion of appropriate description of randomization, allocation concealment, blinding and dropout were 78.43% (40/51), 50.98% (26/51), 52.94% (27/51) and 88.24% (45/51) respectively. Additionally, 90.20% (46/51) trials were used intention-to-treat analysis.
Supplementary Table 2: Statistical inconsistency between direct and indirect comparisons was generally low for weight control. Most loops (90.70%, 39/43) of 27 triangular loops and 16 quadratic loops were consistent.
Supplementary Figure 1: Probabilities for each treatment can be plotted in absolute rankograms or cumulative rankograms. A simple numerical summary supplying the graphical display of cumulative ranking used to estimate the surface under the cumulative line for each treatment. Larger SUCRA means the drug has lower ranking.
Supplementary Figure 2: The forest plots for inconsistence check showed that most loops' 95%CIs included 0, meaning the direct estimate of the summary effect was not different from the indirect estimate.

## Figures and Tables

**Table 1:** Characteristics of the studies included in the network meta-analysis.

Number	Study ID	Investigational treatments	Size	Therapy duration	Baseline information		
					Age	Course of T2D	HbA_1c_
1	Apovian CM, 2010	EX10BID, placebo	142	24	54.8	5.5	7.6
2	Arnolds S, 2010	EX10BID, placebo, and sitagliptin	48	4	56.5	5.5	8.2
3	Barnett AH, 2007	EX10BID, insulin	276	16	54.9	7.4	9
4	Bergenstal R, 2009	EX10BID, insulin	248	24	52.2	8.5	10.2
5	Bunck MC, 2009	EX10BID, insulin	69	52	58.3	4.9	7.5
6	Buse JB, 2004	EX5BID, EX10BID, and placebo	377	30	55	6.3	8.6
7	Buse JB, 2011	EX10BID, placebo	259	30	59	12	8.4
8	DURATION-1, Drucker DJ, 2008	EX10BID, EX2QW	295	30	55	6.5	8.3
9	DURATION-2, Bergenstal RM, 2010	EX2QW, sitagliptin, and TZD	491	26	52.5	6	8.6
10	DURATION-3, Diamant M, 2012	EX2QW, insulin	466	84	58	7.9	8.3
11	DURATION-4, Russell-JonesD, 2012	EX2QW, sitagliptin, Met, and TZD	820	26	53.8	2.7	8.5
12	DURATION-6, Buse JB, 2013	EX2QW, LIR1.8	802	26	57	—	8.5
13	Davies M, 2013	EX2QW, insulin	194	26	58.5	—	8.4
14	Davies MJ, 2009	EX10BID, insulin	204	26	56.5	8.7	8.6
15	Davis SN, 2007	EX10BID, insulin	45	16	53	11	8.1
16	DeFronzo RA, 2005	EX5BID, EX10BID, and placebo	336	30	53	5.8	8.2
17	DeFronzo RA, 2010	EX10BID, TZD	88	20	56	4.7	7.8
18	Derosa G, 2010	EX10BID, SU	116	52	56.5	—	8.9
19	Derosa G, 2011	EX10BID, SU	101	52	55.5	—	8.8
20	Derosa G, 2012	EX10BID, placebo	171	48	57.0	7.7	8.0
21	Forst T, 2012	LIR1.8, placebo	40	12	55.1	3.8	6.3
22	Gallwitz B, 2011	EX10BID, insulin	354	26	57	5	7.9
23	Gallwitz B, EUREXA, 2012	EX10BID, SU	386	234	56	5.8	—
24	Gao Y, 2009	EX10BID, placebo	466	16	54.5	8	8.3
25	Harder H, 2004	LIR0.6, placebo	33	8	60	4.1	7.5
26	Heine RJ, 2005	EX10BID, insulin	535	26	58.9	9.6	8.2
27	Inagaki N, 2012	EX2QW, insulin	426	26	56.76	9.03	8.5
28	Iwamoto K, 2009	EX2QW, placebo	19	10	58	6	7.4
29	Ji LN, 2013	EX10BID, EX2QW	574	26	55	—	—
30	Kadowaki T, 2009	EX5BID, placebo, and EX10BID	114	12	60.3	11.8	8
31	Kadowaki T, 2011	EX5BID, EX10BID, and placebo	178	24	58.4	12	8.2
32	Kaku K, 2010	LIR0.9, LIR0.6, and placebo	264	52	59.7	10.3	8.4
33	Kendall DM, 2005	EX5BID, EX10BID, and placebo	733	30	55.3	8.9	8.5
34	Kim D, 2007	EX2QW, placebo	29	15	54	5	8.5
35	LEAD-1 Marre M, 2009	LIR1.2, LIR0.6, LIR1.8, TZD, and placebo	1041	26	56.1	6.5	8.4
36	LEAD-2 Nauck M, 2009	LIR1.2, LIR0.6, LIR1.8, SU, and placebo	1077	104	57	7.9	8.4
37	LEAD-3 Garder A, 2011	LIR1.2, LIR1.8, and SU	733	104	53	5.4	8.3
38	LEAD-4 Zinman B, 2009	LIR1.2, LIR1.8, and placebo	533	26	55	9	8.5
39	LEAD-5 Russell-Jones D, 2009	LIR1.8, insulin, and placebo	576	26	57.6	9.4	8.3
40	LEAD-6-Buse JB, 2009	EX10BID, LIR1.8	464	26	56.7	8.2	—
41	Liutkus J, 2010	EX10BID, placebo	165	26	54.7	6.4	8.2
42	Moretto TJ, 2008	EX5BID, EX10BID, and placebo	232	24	54	2	7.8
43	NCT00620282, 2011	LIR1.8, SU, and placebo	47	12	58.5	6.8	7.2
44	NCT00701935, 2013	EX10BID, placebo	75	24	58.2	57.9	—
45	Nauck MA, 2007	EX10BID, insulin	501	52	58.5	9.9	8.6
46	Pratley R, 2011	LIR1.2, LIR1.8, and sitagliptin	644	52	55.3	6.2	8.4
47	Seino Y, 2008	LIR0.6, LIR0.9, and placebo	135	14	57	8	8.3
48	Seino Y, 2010	LIR0.9, SU	400	52	58.3	8.3	8.9
49	Yang W, 2011	LIR0.6, LIR1.2, LIR1.8, and SU	907	16	53.3	7.5	8.5
50	Yuan GH, 2012	EX10BID, Met	59	26	50.5	<1 month	8.2
51	Zinman B, 2007	EX10BID, and placebo	233	16	56	8	7.9

Note: LEAD: liraglutide effect and action in diabetes; DURATION: diabetes therapy utilization: researching changes in HbA_1c_, weight, and other factors through intervention with exenatide once weekly; EX5BID: exenatide 5 *μ*g twice daily; EX10BID: exenatide 10 *μ*g twice daily; EX2QW: exenatide 2 mg once weekly; LIR0.6: liraglutide 0.6 mg once daily; LIR0.9: liraglutide 0.9 mg once daily; LIR1.2: liraglutide 1.2 mg once daily; LIR1.8: liraglutide 1.8 mg once daily; SU: sulfonylureas; TZD: thiazolidinediones; Met: metformin. —: unavailable information.

**Table 2:** Results of network meta-analysis (data under the cells marked with underlined data) and direct comparison (data above the cells marked with underlined data) of all treatments.

EX10BID	−0.61 kg (−1.37, 0.15)	**−0.66 **kg **(−1.11, −0.21)**	**−4.77** kg **(−5.48, −4.05)**	—	—	—	0.37 kg (−0.55, 1.29)	**−1.99** kg **(−2.77, −1.21)**	**−7.30** kg **(−12.82, −1.78)**	−1.00 kg (−2.14, 0.14)	**−4.30** kg **(−5.82, −2.78)**	**−1.38** kg **(−1.74, −1.03)**
−1.00 kg (−2.01, 0.03)	EX2QW	—	**−3.17** kg **(−5.48, −0.85)**	—	—	—	**0.89** kg **(0.39, 1.39)**	0.00 kg (−1.25, 1.25)	—	**−1.38** kg **(−2.06, −0.70)**	**−4.33** kg **(−5.90, −2.77)**	−1.27 kg (−5.72, 3.18)
**−1.07 **kg **(−2.14, −0.02)**	−0.07 kg (−1.47, 1.32)	EX5BID	—	—	—	—	—	—	—	—	—	−0.50 kg (−1.09, 0.09)
**−4.52 **kg **(−5.39, −3.65)**	**−3.53 **kg **(−4.62, −2.44)**	**−3.46 **kg **(−4.78, −2.11)**	Insulin	—	—	—	**3.40** kg **(2.49, 4.31)**	—	—	—	—	**2.02** kg **(1.02, 3.02)**
**−2.38 **kg **(−3.71, −1.06)**	−1.38 kg (−2.91, 0.13)	−1.31 kg (−2.91, 0.29)	**2.14** kg **(0.63, 3.65)**	LIR0.6QD	0.70 kg (−3.19, 4.59)	**0.54** kg **(0.23, 0.85)**	**0.74** kg **(0.42, 1.05)**	—	**−2.27** kg **(−3.11, −1.42)**	—	**−1.40** kg **(−1.95, −0.85)**	0.33 kg (−0.22, 0.89)
−2.48 kg (−5.65, 0.53)	−1.48 kg (−4.74, 1.67)	−1.40 kg (−4.70, 1.77)	2.04 kg (−1.19, 5.16)	−0.10 kg (−3.24, 2.95)	LIR0.9QD	—	—	—	−2.11 kg (−6.01, 1.79)	—	—	0.49 kg (−3.63, 4.60)
**−1.62 **kg **(−2.79, −0.43)**	−0.61 kg (−2.01, 0.75)	−0.55 kg (−2.03, 0.93)	**2.91** kg **(1.51, 4.29)**	0.76 kg (−0.60, 2.12)	0.87 kg (−2.24, 4.11)	LIR1.2QD	**0.46** kg **(0.11, 0.81)**	—	**−2.94** kg **(−3.74, −2.14)**	**−1.62** kg **(−2.48, −0.76)**	**−1.80** kg **(−2.35, −1.25)**	−0.79 kg (−2.06, 0.48)
−0.95 kg (−1.93, 0.03)	0.06 kg (−1.14, 1.26)	0.12 kg (−1.24, 1.47)	**3.58** kg **(2.39, 4.78)**	**1.43** kg **(0.15, 2.73)**	1.53 kg (−1.50, 4.73)	0.68 kg (−0.40, 1.74)	LIR1.8QD	—	**−3.12** kg **(−3.80, −2.44)**	**−2.52** kg **(−3.38, −1.66)**	**−2.30** kg **(−2.85, −1.75)**	**−1.32** kg **(−2.22, −0.43)**
−1.44 kg (−3.32, 0.45)	−0.43 kg (−2.41, 1.53)	−0.37 kg (−2.52, 1.74)	**3.10** kg **(1.06, 5.11)**	0.95 kg (−1.32, 3.18)	1.06 kg (−2.50, 4.66)	0.17 kg (−1.97, 2.33)	−0.49 kg (−2.55, 1.51)	Met	—	**−1.20** kg **(−2.26, −0.14)**	**−3.50** kg **(−4.56, −2.44)**	—
**−5.30 **kg **(−6.38, −4.24)**	**−4.29 **kg **(−5.66, −2.91)**	**−4.23** kg **(−5.65, −2.79)**	−0.77 kg (−2.12, 0.55)	**−2.92** kg **(−4.27, −1.53)**	−2.82 kg (−5.85, 0.29)	**−3.68** kg **(−4.92, −2.48)**	**−4.35** kg **(−5.45, −3.24)**	**−3.87** kg **(−5.99, −1.73)**	SU	—	—	**2.03** kg **(0.91, 3.15)**
**−2.21 **kg **(−3.59, −0.83)**	−1.20 kg (−2.64, 0.23)	−1.14 kg (−2.83, 0.55)	**2.31** kg **(0.76, 3.85)**	0.16 kg (−1.55, 1.93)	0.27 kg (−3.01, 3.61)	−0.59 kg (−2.17, 0.97)	−1.26 kg (−2.72, 0.19)	−0.77 kg (−2.87, 1.32)	**3.09** kg **(1.49, 4.69)**	Sitagliptin	**−2.94** kg **(−4.21, −1.67)**	−0.30 kg (−1.37, 0.77)
**−4.59 **kg **(−5.99, −3.24)**	**−3.59 **kg **(−5.04, −2.16)**	**−3.53** kg **(−5.23, −1.85)**	−0.07 kg (−1.62, 1.47)	**−2.21** kg **(−3.92, −0.53)**	−2.11 kg (−5.31, 1.23)	**−2.98** kg **(−4.56, −1.41)**	**−3.65** kg **(−5.13, −2.19)**	**−3.16** kg **(−5.26, −1.09)**	0.70 kg (−0.92, 2.27)	**−2.38** kg **(−3.95, −0.80)**	TZD	**2.20** kg **(1.54, 2.86)**
**−1.92 **kg **(−2.61, −1.24)**	−0.92 kg (−2.01, 0.13)	−0.86 kg (−1.90, 0.20)	**2.60** kg **(1.56, 3.62)**	0.46 kg (−0.80, 1.71)	0.56 kg (−2.48, 3.68)	−0.31 kg (−1.44, 0.81)	**−0.98** kg **(−1.94, −0.02)**	−0.49 kg (−2.45, 1.46)	**3.37** kg **(2.29, 4.48)**	0.28 kg (−1.09, 1.67)	**2.67** kg **(1.28, 4.07)**	Placebo

Treatments are reported in alphabetical order. For data above the cells marked with underlined data, comparisons between treatments should be read from left to right and the estimate is in the cell in common between the column-defining treatment and the row-defining treatment; for data under the cells marked with underlined data, it is reverse order to read the results. EX5BID: exenatide 5 *μ*g twice daily; EX10BID: exenatide 10 *μ*g twice daily; EX2QW: exenatide 2 mg once weekly; LIR0.6: liraglutide 0.6 mg once daily; LIR0.9: liraglutide 0.9 mg once daily; LIR1.2: liraglutide 1.2 mg once daily; LIR1.8: liraglutide 1.8 mg once daily; SU: sulfonylureas; TZD: thiazolidinediones; Met: metformin.

**Table 3:** Mean differences of weight and the rank of GLP-1 RAs on weight loss in comparison with placebo.

Treatment	Weight control		
	MD (95% CI)	SUCRA	Rank
EX10BID	−1.92 (−2.61, −1.24)	0.98298	1
EX2QW	−0.92 (−2.01, 0.13)	0.76228	3
EX5BID	−0.86 (−1.90, 0.20)	0.75020	4
LIR0.6	0.46 (−0.80, 1.71)	0.38829	10
LIR0.9	0.56 (−2.48, 3.68)	0.42898	8
LIR1.2	−0.31 (−1.44, 0.81)	0.58441	6
LIR1.8	−0.98 (−1.94, −0.02)	0.78891	2
Insulin	2.60 (1.56, 3.62)	0.12863	11
Met	−0.49 (−2.45, 1.46)	0.63318	5
SU	3.37 (2.29, 4.48)	0.03021	13
Sitagliptin	0.28 (−1.09, 1.67)	0.42465	9
TZD	2.67 (1.28, 4.07)	0.11287	12
Placebo	Reference	0.48504	7

Note: EX5BID: exenatide 5 *μ*g twice daily; EX10BID: exenatide 10 *μ*g twice daily; EX2QW: exenatide 2 mg once weekly; LIR0.6: liraglutide 0.6 mg once daily; LIR0.9: liraglutide 0.9 mg once daily; LIR1.2: liraglutide 1.2 mg once daily; LIR1.8: liraglutide 1.8 mg once daily; SU: sulfonylureas; TZD: thiazolidinediones; Met: metformin.
