# Supplementary material for: Effects of Glucagon-Like Peptide-1 Receptor Agonists on Weight Loss in Patients with Type 2 Diabetes: A Systematic Review and Network Meta-Analysis
Source: J Diabetes Res. 2015 Jan 20;2015:157201. doi: 10.1155/2015/157201 (PMC4320855; doi:10.1155/2015/157201)
Supplement: Supplementary file 1 — Supplementary Table 1: The overall quality of total 51 studies was rated according to JADAD scale. The proportion of appropriate description of randomization, allocation concealment, blinding and dropout were 78.43% (40/51), 50.98% (26/51), 52.94% (27/51) and 88.24% (45/51) respectively. Additionally, 90.20% (46/51) trials were used intention-to-treat analysis. Supplementary Table 2: Statistical inconsistency between direct and indirect comparisons was generally low for weight control. Most loops (90.70%, 39/43) of 27 triangular loops and 16 quadratic loops were consistent. Supplementary Figure 1: Probabilities for each treatment can be plotted in absolute rankograms or cumulative rankograms. A simple numerical summary supplying the graphical display of cumulative ranking used to estimate the surface under the cumulative line for each treatment. Larger SUCRA means the drug has lower ranking. Supplementary Figure 2: The forest plots for inconsistence check showed that most loops' 95%CIs included 0, meaning the direct estimate of the summary effect was not different from the indirect estimate. [file 157201.f1.pdf]

```
model {
	for (i in 1:ns) {
		# Likelihood for each arm
		for (k in 1:na[i]) {
			m[i, k] ~ dnorm(theta[i, k], prec[i, k])
			theta[i, k] <- mu[i] + delta[i, k]
			prec[i, k] <- pow(e[i, k], -2)
		}

		# Study-level relative effects
		# The arms are given in the order (arm_1, arm_2, ..., arm_{n_a-1}, arm_{n_a}).
		# The relative effects are parameterized as d[arm_1, arm_k].
		w[i, 1] <- 0
		delta[i, 1] <- 0
		for (k in 2:na[i]) { # parameterize multi-arm trials using a trick to avoid dmnorm
			delta[i, k] ~ dnorm(md[i, k], taud[i, k])
			md[i, k] <- d[t[i, 1], t[i, k]] + sw[i, k]
			taud[i, k] <- tau.d * 2 * (k - 1) / k
			w[i, k] <- delta[i, k] - d[t[i, 1], t[i, k]]
			sw[i, k] <- sum(w[i, 1:k-1]) / (k - 1)
		}
	}

	# Relative effect matrix
	d[1,1] <- 0
	d[1,2] <- d.EX10BID.EX2QW
	d[1,3] <- d.EX10BID.Exe5ug
	d[1,4] <- d.EX10BID.Insulin
	d[1,5] <- d.EX10BID.SU + d.SU.LIR06
	d[1,6] <- d.EX10BID.SU + d.SU.LIR09
	d[1,7] <- d.EX10BID.SU + d.SU.LIR12
	d[1,8] <- d.EX10BID.LIR18
	d[1,9] <- d.EX10BID.Met
	d[1,10] <- d.EX10BID.SU
	d[1,11] <- d.EX10BID.Sitagliptin
	d[1,12] <- d.EX10BID.TZD
	d[1,13] <- d.EX10BID.placebo
	d[2,1] <- -d.EX10BID.EX2QW
	d[2,2] <- 0
	d[2,3] <- -d.EX10BID.EX2QW + d.EX10BID.Exe5ug
	d[2,4] <- -d.EX10BID.EX2QW + d.EX10BID.Insulin
	d[2,5] <- -d.EX10BID.EX2QW + d.EX10BID.SU + d.SU.LIR06
	d[2,6] <- -d.EX10BID.EX2QW + d.EX10BID.SU + d.SU.LIR09
	d[2,7] <- -d.EX10BID.EX2QW + d.EX10BID.SU + d.SU.LIR12
```

```
d[2,8] <- -d.EX10BID.EX2QW + d.EX10BID.LIR18
d[2,9] <- -d.EX10BID.EX2QW + d.EX10BID.Met
d[2,10] <- -d.EX10BID.EX2QW + d.EX10BID.SU
d[2,11] <- -d.EX10BID.EX2QW + d.EX10BID.Sitagliptin
d[2,12] <- -d.EX10BID.EX2QW + d.EX10BID.TZD
d[2,13] <- -d.EX10BID.EX2QW + d.EX10BID.placebo
d[3,1] <- -d.EX10BID.Exe5ug
d[3,2] <- d.EX10BID.EX2QW + -d.EX10BID.Exe5ug
d[3,3] <- 0
d[3,4] <- -d.EX10BID.Exe5ug + d.EX10BID.Insulin
d[3,5] <- -d.EX10BID.Exe5ug + d.EX10BID.SU + d.SU.LIR06
d[3,6] <- -d.EX10BID.Exe5ug + d.EX10BID.SU + d.SU.LIR09
d[3,7] <- -d.EX10BID.Exe5ug + d.EX10BID.SU + d.SU.LIR12
d[3,8] <- -d.EX10BID.Exe5ug + d.EX10BID.LIR18
d[3,9] <- -d.EX10BID.Exe5ug + d.EX10BID.Met
d[3,10] <- -d.EX10BID.Exe5ug + d.EX10BID.SU
d[3,11] <- -d.EX10BID.Exe5ug + d.EX10BID.Sitagliptin
d[3,12] <- -d.EX10BID.Exe5ug + d.EX10BID.TZD
d[3,13] <- -d.EX10BID.Exe5ug + d.EX10BID.placebo
d[4,1] <- -d.EX10BID.Insulin
d[4,2] <- d.EX10BID.EX2QW + -d.EX10BID.Insulin
d[4,3] <- d.EX10BID.Exe5ug + -d.EX10BID.Insulin
d[4,4] <- 0
d[4,5] <- -d.EX10BID.Insulin + d.EX10BID.SU + d.SU.LIR06
d[4,6] <- -d.EX10BID.Insulin + d.EX10BID.SU + d.SU.LIR09
d[4,7] <- -d.EX10BID.Insulin + d.EX10BID.SU + d.SU.LIR12
d[4,8] <- -d.EX10BID.Insulin + d.EX10BID.LIR18
d[4,9] <- -d.EX10BID.Insulin + d.EX10BID.Met
d[4,10] <- -d.EX10BID.Insulin + d.EX10BID.SU
d[4,11] <- -d.EX10BID.Insulin + d.EX10BID.Sitagliptin
d[4,12] <- -d.EX10BID.Insulin + d.EX10BID.TZD
d[4,13] <- -d.EX10BID.Insulin + d.EX10BID.placebo
d[5,1] <- -d.EX10BID.SU + -d.SU.LIR06
d[5,2] <- d.EX10BID.EX2QW + -d.EX10BID.SU + -d.SU.LIR06
d[5,3] <- d.EX10BID.Exe5ug + -d.EX10BID.SU + -d.SU.LIR06
d[5,4] <- d.EX10BID.Insulin + -d.EX10BID.SU + -d.SU.LIR06
d[5,5] <- 0
d[5,6] <- -d.SU.LIR06 + d.SU.LIR09
d[5,7] <- -d.SU.LIR06 + d.SU.LIR12
d[5,8] <- d.EX10BID.LIR18 + -d.EX10BID.SU + -d.SU.LIR06
d[5,9] <- d.EX10BID.Met + -d.EX10BID.SU + -d.SU.LIR06
d[5,10] <- -d.SU.LIR06
d[5,11] <- -d.EX10BID.SU + d.EX10BID.Sitagliptin + -d.SU.LIR06
d[5,12] <- -d.EX10BID.SU + d.EX10BID.TZD + -d.SU.LIR06
```

```
d[5,13] <- -d.EX10BID.SU + d.EX10BID.placebo + -d.SU.LIR06
d[6,1] <- -d.EX10BID.SU + -d.SU.LIR09
d[6,2] <- d.EX10BID.EX2QW + -d.EX10BID.SU + -d.SU.LIR09
d[6,3] <- d.EX10BID.Exe5ug + -d.EX10BID.SU + -d.SU.LIR09
d[6,4] <- d.EX10BID.Insulin + -d.EX10BID.SU + -d.SU.LIR09
d[6,5] <- d.SU.LIR06 + -d.SU.LIR09
d[6,6] <- 0
d[6,7] <- -d.SU.LIR09 + d.SU.LIR12
d[6,8] <- d.EX10BID.LIR18 + -d.EX10BID.SU + -d.SU.LIR09
d[6,9] <- d.EX10BID.Met + -d.EX10BID.SU + -d.SU.LIR09
d[6,10] <- -d.SU.LIR09
d[6,11] <- -d.EX10BID.SU + d.EX10BID.Sitagliptin + -d.SU.LIR09
d[6,12] <- -d.EX10BID.SU + d.EX10BID.TZD + -d.SU.LIR09
d[6,13] <- -d.EX10BID.SU + d.EX10BID.placebo + -d.SU.LIR09
d[7,1] <- -d.EX10BID.SU + -d.SU.LIR12
d[7,2] <- d.EX10BID.EX2QW + -d.EX10BID.SU + -d.SU.LIR12
d[7,3] <- d.EX10BID.Exe5ug + -d.EX10BID.SU + -d.SU.LIR12
d[7,4] <- d.EX10BID.Insulin + -d.EX10BID.SU + -d.SU.LIR12
d[7,5] <- d.SU.LIR06 + -d.SU.LIR12
d[7,6] <- d.SU.LIR09 + -d.SU.LIR12
d[7,7] <- 0
d[7,8] <- d.EX10BID.LIR18 + -d.EX10BID.SU + -d.SU.LIR12
d[7,9] <- d.EX10BID.Met + -d.EX10BID.SU + -d.SU.LIR12
d[7,10] <- -d.SU.LIR12
d[7,11] <- -d.EX10BID.SU + d.EX10BID.Sitagliptin + -d.SU.LIR12
d[7,12] <- -d.EX10BID.SU + d.EX10BID.TZD + -d.SU.LIR12
d[7,13] <- -d.EX10BID.SU + d.EX10BID.placebo + -d.SU.LIR12
d[8,1] <- -d.EX10BID.LIR18
d[8,2] <- d.EX10BID.EX2QW + -d.EX10BID.LIR18
d[8,3] <- d.EX10BID.Exe5ug + -d.EX10BID.LIR18
d[8,4] <- d.EX10BID.Insulin + -d.EX10BID.LIR18
d[8,5] <- -d.EX10BID.LIR18 + d.EX10BID.SU + d.SU.LIR06
d[8,6] <- -d.EX10BID.LIR18 + d.EX10BID.SU + d.SU.LIR09
d[8,7] <- -d.EX10BID.LIR18 + d.EX10BID.SU + d.SU.LIR12
d[8,8] <- 0
d[8,9] <- -d.EX10BID.LIR18 + d.EX10BID.Met
d[8,10] <- -d.EX10BID.LIR18 + d.EX10BID.SU
d[8,11] <- -d.EX10BID.LIR18 + d.EX10BID.Sitagliptin
d[8,12] <- -d.EX10BID.LIR18 + d.EX10BID.TZD
d[8,13] <- -d.EX10BID.LIR18 + d.EX10BID.placebo
d[9,1] <- -d.EX10BID.Met
d[9,2] <- d.EX10BID.EX2QW + -d.EX10BID.Met
d[9,3] <- d.EX10BID.Exe5ug + -d.EX10BID.Met
d[9,4] <- d.EX10BID.Insulin + -d.EX10BID.Met
```

```
d[9,5] <- -d.EX10BID.Met + d.EX10BID.SU + d.SU.LIR06
d[9,6] <- -d.EX10BID.Met + d.EX10BID.SU + d.SU.LIR09
d[9,7] <- -d.EX10BID.Met + d.EX10BID.SU + d.SU.LIR12
d[9,8] <- d.EX10BID.LIR18 + -d.EX10BID.Met
d[9,9] <- 0
d[9,10] <- -d.EX10BID.Met + d.EX10BID.SU
d[9,11] <- -d.EX10BID.Met + d.EX10BID.Sitagliptin
d[9,12] <- -d.EX10BID.Met + d.EX10BID.TZD
d[9,13] <- -d.EX10BID.Met + d.EX10BID.placebo
d[10,1] <- -d.EX10BID.SU
d[10,2] <- d.EX10BID.EX2QW + -d.EX10BID.SU
d[10,3] <- d.EX10BID.Exe5ug + -d.EX10BID.SU
d[10,4] <- d.EX10BID.Insulin + -d.EX10BID.SU
d[10,5] <- d.SU.LIR06
d[10,6] <- d.SU.LIR09
d[10,7] <- d.SU.LIR12
d[10,8] <- d.EX10BID.LIR18 + -d.EX10BID.SU
d[10,9] <- d.EX10BID.Met + -d.EX10BID.SU
d[10,10] <- 0
d[10,11] <- -d.EX10BID.SU + d.EX10BID.Sitagliptin
d[10,12] <- -d.EX10BID.SU + d.EX10BID.TZD
d[10,13] <- -d.EX10BID.SU + d.EX10BID.placebo
d[11,1] <- -d.EX10BID.Sitagliptin
d[11,2] <- d.EX10BID.EX2QW + -d.EX10BID.Sitagliptin
d[11,3] <- d.EX10BID.Exe5ug + -d.EX10BID.Sitagliptin
d[11,4] <- d.EX10BID.Insulin + -d.EX10BID.Sitagliptin
d[11,5] <- d.EX10BID.SU + -d.EX10BID.Sitagliptin + d.SU.LIR06
d[11,6] <- d.EX10BID.SU + -d.EX10BID.Sitagliptin + d.SU.LIR09
d[11,7] <- d.EX10BID.SU + -d.EX10BID.Sitagliptin + d.SU.LIR12
d[11,8] <- d.EX10BID.LIR18 + -d.EX10BID.Sitagliptin
d[11,9] <- d.EX10BID.Met + -d.EX10BID.Sitagliptin
d[11,10] <- d.EX10BID.SU + -d.EX10BID.Sitagliptin
d[11,11] <- 0
d[11,12] <- -d.EX10BID.Sitagliptin + d.EX10BID.TZD
d[11,13] <- -d.EX10BID.Sitagliptin + d.EX10BID.placebo
d[12,1] <- -d.EX10BID.TZD
d[12,2] <- d.EX10BID.EX2QW + -d.EX10BID.TZD
d[12,3] <- d.EX10BID.Exe5ug + -d.EX10BID.TZD
d[12,4] <- d.EX10BID.Insulin + -d.EX10BID.TZD
d[12,5] <- d.EX10BID.SU + -d.EX10BID.TZD + d.SU.LIR06
d[12,6] <- d.EX10BID.SU + -d.EX10BID.TZD + d.SU.LIR09
d[12,7] <- d.EX10BID.SU + -d.EX10BID.TZD + d.SU.LIR12
d[12,8] <- d.EX10BID.LIR18 + -d.EX10BID.TZD
d[12,9] <- d.EX10BID.Met + -d.EX10BID.TZD
```

```
        d[12,10] <- d.EX10BID.SU + -d.EX10BID.TZD
        d[12,11] <- d.EX10BID.Sitagliptin + -d.EX10BID.TZD
        d[12,12] <- 0
        d[12,13] <- -d.EX10BID.TZD + d.EX10BID.placebo
        d[13,1] <- -d.EX10BID.placebo
        d[13,2] <- d.EX10BID.EX2QW + -d.EX10BID.placebo
        d[13,3] <- d.EX10BID.Exe5ug + -d.EX10BID.placebo
        d[13,4] <- d.EX10BID.Insulin + -d.EX10BID.placebo
        d[13,5] <- d.EX10BID.SU + -d.EX10BID.placebo + d.SU.LIR06
        d[13,6] <- d.EX10BID.SU + -d.EX10BID.placebo + d.SU.LIR09
        d[13,7] <- d.EX10BID.SU + -d.EX10BID.placebo + d.SU.LIR12
        d[13,8] <- d.EX10BID.LIR18 + -d.EX10BID.placebo
        d[13,9] <- d.EX10BID.Met + -d.EX10BID.placebo
        d[13,10] <- d.EX10BID.SU + -d.EX10BID.placebo
        d[13,11] <- d.EX10BID.Sitagliptin + -d.EX10BID.placebo
        d[13,12] <- d.EX10BID.TZD + -d.EX10BID.placebo
        d[13,13] <- 0

        # Study baseline priors
        for (i in 1:ns) {
                mu[i] ~ dnorm(0, 2.938E-5)
        }

        # Variance prior
        sd.d ~ dunif(0, 1.23E1)
        tau.d <- pow(sd.d, -2)

        # Effect parameter priors
        d.EX10BID.EX2QW ~ dnorm(0, 2.938E-5)
        d.EX10BID.Exe5ug ~ dnorm(0, 2.938E-5)
        d.EX10BID.Insulin ~ dnorm(0, 2.938E-5)
        d.EX10BID.LIR18 ~ dnorm(0, 2.938E-5)
        d.EX10BID.Met ~ dnorm(0, 2.938E-5)
        d.EX10BID.SU ~ dnorm(0, 2.938E-5)
        d.EX10BID.Sitagliptin ~ dnorm(0, 2.938E-5)
        d.EX10BID.TZD ~ dnorm(0, 2.938E-5)
        d.EX10BID.placebo ~ dnorm(0, 2.938E-5)
        d.SU.LIR06 ~ dnorm(0, 2.938E-5)
        d.SU.LIR09 ~ dnorm(0, 2.938E-5)
        d.SU.LIR12 ~ dnorm(0, 2.938E-5)
}

list(
ns = 51,
```

```
na = c(2, 3, 2, 2, 2, 3, 2, 2, 3, 2, 4, 2, 2, 2, 2, 3, 2, 2, 2, 2, 2, 2, 2, 2, 2, 2, 2, 2, 3, 3, 3, 3, 2, 5, 5, 3, 3, 3, 2, 2, 3, 3, 2, 2, 3, 3, 2, 4, 2, 2),
t = structure(.Data = c(1, 13, NA, NA, NA, 1, 11, 13, NA, NA, 1, 4, NA, NA, NA, 1, 4, NA, NA, NA, 1, 4, NA, NA, NA, 1, 3, 13, NA, NA, 1, 13, NA, NA, NA, 1, 2, NA, NA, NA, 2, 11, 12, NA, NA, 2, 4, NA, NA, NA, 2, 9, 11, 12, NA, 2, 8, NA, NA, NA, 2, 4, NA, NA, NA, 1, 4, NA, NA, NA, 1, 4, NA, NA, NA, 1, 3, 13, NA, NA, 1, 12, NA, NA, NA, 1, 10, NA, NA, NA, 1, 10, NA, NA, NA, 1, 13, NA, NA, NA, 8, 13, NA, NA, NA, 1, 4, NA, NA, NA, 1, 10, NA, NA, NA, 1, 13, NA, NA, NA, 5, 13, NA, NA, NA, 1, 4, NA, NA, NA, 2, 4, NA, NA, NA, 2, 13, NA, NA, NA, 1, 2, NA, NA, NA, 1, 3, 13, NA, NA, 1, 3, 13, NA, NA, 5, 6, 13, NA, NA, 1, 3, 13, NA, NA, 2, 13, NA, NA, NA, 5, 7, 8, 12, 13, 10, 5, 7, 8, 13, 10, 7, 8, NA, NA, 7, 8, 13, NA, NA, 4, 8, 13, NA, NA, 1, 8, NA, NA, NA, 1, 13, NA, NA, NA, 1, 3, 13, NA, NA, 10, 8, 13, NA, NA, 1, 13, NA, NA, NA, 1, 4, NA, NA, NA, 7, 8, 11, NA, NA, 5, 6, 13, NA, NA, 10, 6, NA, NA, NA, 10, 5, 7, 8, NA, 1, 9, NA, NA, NA, 1, 13, NA, NA, NA), .Dim = c(51, 5)),
m = structure(.Data = c(-6.16, -3.97, NA, NA, NA, -0.9, 0.1, 0.4, NA, NA, -1.6, 0.6, NA, NA, NA, -1.9, 4.1, NA, NA, NA, -3.6, 1.0, NA, NA, NA, -1.6, -0.9, -0.6, NA, NA, -1.78, 0.96, NA, NA, NA, -3.6, -3.7, NA, NA, NA, -2.3, -0.8, 2.8, NA, NA, -2.1, 2.4, NA, NA, NA, -2.0, -2.0, -0.8, 1.5, NA, -2.68, -3.57, NA, NA, NA, -2.79, 0.88, NA, NA, NA, -2.73, 2.98, NA, NA, NA, -4.2, 0.5, NA, NA, NA, -2.8, -1.6, -0.3, NA, NA, -2.82, 1.48, NA, NA, NA, -8.0, 4.3, NA, NA, NA, -5.1, -0.9, NA, NA, NA, -6.4, -2.3, NA, NA, NA, -3.5, -0.2, NA, NA, NA, -4.1, 1.02, NA, NA, NA, -3.92, 1.47, NA, NA, NA, -1.2, -0.1, NA, NA, NA, -0.7, -0.9, NA, NA, NA, -2.3, 1.8, NA, NA, NA, -1.67, -0.34, NA, NA, NA, -0.8, -1.6, NA, NA, NA, -2.45, -1.63, NA, NA, NA, -1.3, -0.2, -0.7, NA, NA, -1.54, -0.39, -0.47, NA, NA, -0.44, -1.37, -1.87, NA, NA, -1.6, -1.6, -0.9, NA, NA, -3.8, -0.04, NA, NA, NA, 0.7, 0.3, -0.2, 2.1, -0.1, 0.7, -2.07, -3.03, -2.91, -1.8, 0.95, -1.89, -2.7, NA, NA, -1.0, -2.0, 0.6, NA, NA, 1.6, -1.8, -0.42, NA, NA, -2.87, -3.24, NA, NA, NA, -2.1, -1.4, NA, NA, NA, -3.1, -2.8, -1.4, NA, NA, 1.038, -1.821, -0.293, NA, NA, -2.54, -0.33, NA, NA, NA, -2.5, 2.9, NA, NA, NA, -2.78, -3.68, -1.16, NA, NA, -0.1, -0.48, -0.95, NA, NA, 1.21, -0.9, NA, NA, NA, 0.1, -1.8, -2.3, -2.4, NA, -5.8, -3.81, NA, NA, NA, -1.75, -0.24, NA, NA, NA), .Dim = c(51, 5)),
e = structure(.Data = c(0.5402433314191458, 0.5197234841721124, NA, NA, NA, 0.425, 0.4, 0.375, NA, NA, 0.29964229882707955, 0.29964229882707955, NA, NA, NA, 0.3412500738508723, 0.4849343154722923, NA, NA, NA, 0.6, 0.80075721739621, NA, NA, NA, 0.30023375905703775, 0.2996331089849718, 0.30025598834425493, NA, NA, 0.35028663966387197, 0.36033226924922374, NA, NA, NA, 0.5031195202938167, 0.5014169112813397, NA, NA, NA, 0.3059503636212907, 0.3314162744766041, 0.30595008505547855, NA, NA, 0.01310243564160837, 0.01310243564160837, NA, NA, NA, 0.44894544894567345, 0.45076673613578017, 0.2999887523453877, 0.2999887523453877, NA, 0.18010173142800803, 0.1799504398550337, NA, NA, NA, 0.3470110468942836, 0.3505229329596172, NA, NA, NA, 0.31, 0.3098634935183308, NA, NA, NA, 0.5570860145311556, 0.425, NA, NA, NA, 0.5004635019800737, 0.4004542874831488, 0.3000899570143675, NA, NA, 0.5472430904086409, 0.5472430904086409, NA, NA, NA, 0.1562269317698865, 0.24371395369996807, NA, NA, NA, 0.18721131622601486, 0.22285714285714286, NA, NA, NA, 1.2152753540027508, 1.490312245214168, NA, NA, NA, 5.2001323243236985, 5.7606290774898055, NA, NA, NA, 0.22001506728916123, 0.2197226312469449, NA, NA, NA, 0.3352578676747763, 0.3188162183680864, NA, NA, NA, 0.14970198315548047, 0.12474111121673642, NA, NA, NA, 0.47571500071446343, 0.6985938257194472, NA, NA, NA, 0.20020353279963501, 0.19969592268862563, NA, NA, NA,
```

0.17021289582914118, 0.1703270992963744, NA, NA, NA, 0.5, 0.5059644256269407, NA, NA, NA, 0.15971673037682732, 0.15985531484078935, NA, NA, NA, 0.2992061568957503, 0.2992061568957503, 0.1992234925906079, NA, NA, 0.26987908815286565, 0.28008047133374936, 0.3904612656845747, NA, NA, 1.8505822179666984, 1.8367241714035838, 2.0531228985045287, NA, NA, 0.19968855418425757, 0.19996836484498118, 0.19979341557439423, NA, NA, 1.3994379824296133, 0.7002244538105519, NA, NA, NA, 0.19653653462412551, 0.20000438591682163, 0.2000384578409477, 0.20024231011107688, 0.2697367137355279, 0.2897081318978874, 0.28027141508848613, 0.29032127148723724, 0.28085228911076227, 0.28, 0.29972029972044956, 0.30985513402497084, 0.32016662328647977, NA, NA, 0.29981267559834457, 0.29981267559834457, 0.29990676517312875, NA, NA, 0.3302356786422022, 0.3296902366978935, 0.3896196976179847, NA, NA, 0.330291750869804, 0.3301813781685309, NA, NA, NA, 0.3995955162118021, 0.49942374200079237, NA, NA, NA, 0.3000534140483178, 0.2997163160888478, 0.2997163160888478, NA, NA, 0.44141483756612604, 0.455, 0.4864154602806124, NA, NA, 0.5693793613601191, 0.720681495656589, NA, NA, NA, 0.19992488708089423, 0.20002520002530003, NA, NA, NA, 0.31030742445906545, 0.3096644249421725, 0.31030742445906545, NA, NA, 2.01991473967481, 2.3095768994656694, 2.27945264215471, NA, NA, 1.6380707425431167, 1.1276239714086973, NA, NA, NA, 0.1447493728911492, 0.14601912855199384, 0.16, 0.17371980724307587, NA, 0.2889689088864584, 0.27064026649069395, NA, NA, NA, 0.2381818181818182, 0.24851164100356687, NA, NA, NA), .Dim = c(51, 5))
)

list(
d.EX10BID.EX2QW = 0.8957255455674726,
d.EX10BID.Exe5ug = 0.4566936431170917,
d.EX10BID.Insulin = 2.5568246591804566,
d.EX10BID.LIR18 = -1.4349212241243023,
d.EX10BID.Met = 2.8322171292134874,
d.EX10BID.SU = 15.939635320893302,
d.EX10BID.Sitagliptin = 0.6609065074387261,
d.EX10BID.TZD = 5.246908996501909,
d.EX10BID.placebo = 1.1160183294916104,
d.SU.LIR06 = -0.6310215124302521,
d.SU.LIR09 = -2.7216762292091135,
d.SU.LIR12 = -5.358891905208765,
mu = c(-6.592355031788382, -1.5936212932280829, -0.8887562006414326, -3.07742897571942, -3.9382627376964843, -1.859508024914645, -2.0287778992029737, -3.029186253212147, -0.7074360520925238, -2.123055528763754, -1.4494121063974497, -2.4590951452841527, -3.704508969847302, -1.9793292539813838, -4.309104774317473, -3.511184711979647, -0.8716996902138328, -8.314731499135071, -4.488120102120745, -4.758467164949556, 7.043630261199388, -3.7395985344990885, -2.208152366349621, -1.0324256589050036, 1.6144574804407366, -2.3351038707397556, -2.0044908423628875,

0.9094015797768922, -3.2537038868569486, -1.8946363763965044, -2.450870101833355,
-0.4166629129104134, -2.131476593245068, -4.4412217507973955, 0.9059500821957938,
0.5883828247086859, 1.7685304142918705, -0.9614289495575915, 0.8707348175340992,
-3.3262757135819294, -2.657202963142497, -3.102886065261921, 0.9038106681075522,
-2.695295267899678, -2.35232048442234, -1.432806031613623, -3.7195415858942114,
0.9415611392094134, -0.28134359333658676, -6.270177052399954, -2.2018823076663225),
delta = structure(.Data = c(NA, 5.974046286972138, NA, NA, NA, NA, -0.43140335495599635,
3.5222373605564297, NA, NA, NA, 2.3250672244147, NA, NA, NA, NA, 7.034702463883187, NA,
NA, NA, NA, 4.508817645162357, NA, NA, NA, NA, -0.37380083248883256, 1.138803176910114,
NA, NA, NA, 1.2905387049526664, NA, NA, NA, NA, -1.0008723170693774, NA, NA, NA, NA,
0.7462104420134413, 4.815387358942273, NA, NA, NA, 4.589574516371187, NA, NA, NA, NA,
1.2789893504197, 2.1736344924629063, 3.303338368406676, NA, NA, -0.35288677305178395,
NA, NA, NA, NA, 2.1235933702059864, NA, NA, NA, NA, 7.10238276328593, NA, NA, NA, NA,
4.444142509821387, NA, NA, NA, NA, 2.1720192599074477, 3.812265201026783, NA, NA, NA,
2.4933878498051394, NA, NA, NA, NA, 12.850019159166807, NA, NA, NA, NA,
4.971592911280548, NA, NA, NA, NA, 3.9680611082585826, NA, NA, NA, NA,
-1.8129264132284097, NA, NA, NA, NA, 4.467286155258912, NA, NA, NA, NA,
5.42357572463013, NA, NA, NA, NA, 1.1475586484652656, NA, NA, NA, NA,
2.090727858125325, NA, NA, NA, NA, 4.065055866203564, NA, NA, NA, NA,
0.41986886882962116, NA, NA, NA, NA, -1.0463403782784744, NA, NA, NA, NA,
0.32052528959738164, NA, NA, NA, NA, 1.5267068403889468, -0.3663832695173306, NA, NA,
NA, 2.366238719056464, -0.4154991408867281, NA, NA, NA, -3.2210845781410304,
-3.062517238258335, NA, NA, NA, 0.417080239262147, 1.7340902613216644, NA, NA, NA,
5.60542715733249, NA, NA, NA, NA, -0.6947031543116333, -1.2793917584852608,
2.0017109141867775, -0.9452102442477434, NA, -3.2671433328744763, -3.1684346328208446,
-3.75231063105181, -1.5772255316517874, NA, -3.0768227602827434, -4.406944301915749,
NA, NA, NA, -3.1892415391059354, 4.074022452729643, NA, NA, NA, -3.183607152034051,
-2.053944577085453, NA, NA, NA, -0.9232742803334055, NA, NA, NA, NA, 2.996012729204016,
NA, NA, NA, NA, 1.782780799565401, 1.8746863192800436, NA, NA, NA, -4.7415427765758835,
-2.4427558315842726, NA, NA, NA, -0.47172188408558613, NA, NA, NA, NA,
6.831275572249652, NA, NA, NA, NA, -0.04660997987276372, 0.27287396614809434, NA, NA,
NA, 2.637900206720261, 0.0808645297814139, NA, NA, NA, 0.5322010424629218, NA, NA, NA,
NA, -1.8909822308236661, -2.441860584598912, -3.0146750817824755, NA, NA,
1.4061196406765075, NA, NA, NA, NA, 1.8698599129299833, NA, NA, NA), .Dim = c(51, 5)),
sd.d = 10.428131099744308
)

list(
d.EX10BID.EX2QW = 0.09744517865831037,
d.EX10BID.Exe5ug = 0.7983981729310514,
d.EX10BID.Insulin = 4.305688424513304,
d.EX10BID.LIR18 = -1.383034021875909,
d.EX10BID.Met = 2.085768806501968,
d.EX10BID.SU = -5.678680258255404,

d.EX10BID.Sitagliptin = 1.7972855302803126,
d.EX10BID.TZD = 5.87486474238661,
d.EX10BID.placebo = 1.2797149494081057,
d.SU.LIR06 = -1.1709950115421972,
d.SU.LIR09 = -5.944330524940246,
d.SU.LIR12 = -2.7317490999450103,
mu = c(-5.688057175038192, -2.277691467473313, -2.050512150948088, -2.1614680258886585, -2.1864688179033713, -1.5333681169578004, -0.9856526020583781, -4.666305703251879, -2.3770224668455593, -2.0674761383467573, -2.2247072744293543, -2.4221325585590807, -2.6149857339819262, -2.210749798703347, -4.759043438179661, -2.9474457005063504, -3.0450201015106937, -7.969903114193894, -5.255837030911234, -9.902111479942226, -0.5183144744236641, -3.95854791050871, -6.30118680935726, -1.5980344731441227, -0.9398021969825163, -0.8925688109056849, -2.535182663172589, -3.0564820738486924, -2.4134918449854417, 0.07436049059740846, -0.6308248287487918, -12.11007529451518, -2.1671069430049785, -6.530072022422267, 0.02926555549167975, 1.5091730680595998, 1.0247476854661062, -1.4840321592518053, 1.3855177583924103, -3.478519467584607, -2.0383920073286284, -3.129650856217219, 1.1611847504449626, -1.778100228781819, -2.1712190941224923, -3.273369909682191, -5.507412044168503, -0.4481437963792916, -0.5024688564062397, -6.802495534449828, -2.4306152089344835),
delta = structure(.Data = c(NA, 2.619606778397892, NA, NA, NA, NA, 0.7744221947372834, 4.818656611488761, NA, NA, NA, 2.314104738969365, NA, NA, NA, NA, 4.230430756087413, NA, NA, NA, NA, 3.9631382376311564, NA, NA, NA, NA, 0.10340684317788729, 0.8309043888598031, NA, NA, NA, 2.4670576241599553, NA, NA, NA, NA, -1.1183803987291088, NA, NA, NA, NA, 4.021626273346377, 4.861004920462494, NA, NA, NA, 4.47774465468012, NA, NA, NA, NA, -1.4822993399863398, 1.2287640914881106, 4.009581213308806, NA, NA, -0.5667449179968562, NA, NA, NA, NA, 3.8769506398719766, NA, NA, NA, NA, 6.557107491858457, NA, NA, NA, NA, -0.4090817387829846, NA, NA, NA, NA, 0.40459803590095245, 3.786655430993033, NA, NA, NA, 6.446144842076464, NA, NA, NA, NA, 12.440072073410263, NA, NA, NA, NA, 2.972601231397289, NA, NA, NA, NA, 0.8411118822718477, NA, NA, NA, NA, 22.73405866725939, NA, NA, NA, NA, 5.669204691131996, NA, NA, NA, NA, 3.0538247800818787, NA, NA, NA, NA, 0.5595780599073762, NA, NA, NA, NA, 0.7351087031086919, NA, NA, NA, NA, 3.5535254928801034, NA, NA, NA, NA, 1.4551482309367794, NA, NA, NA, NA, -1.8546955321628238, NA, NA, NA, NA, 1.1103679769125623, NA, NA, NA, NA, 1.1243208042220605, 0.8886960638517654, NA, NA, NA, 1.1704782869588939, -0.2732695682384423, NA, NA, NA, -5.2278313152858775, -4.570362415238386, NA, NA, NA, 0.5630757383518895, 1.0006241700451697, NA, NA, NA, 4.122517987542122, NA, NA, NA, NA, -0.12513959334269853, -1.3311643405882503, 1.0578454661138423, -0.21004427562716044, NA, -2.5232930044954025, -3.173887303227459, -3.9125920147465307, -2.6139135431088816, NA, -4.001000550690312, -3.9227172519334603, NA, NA, NA, -0.6167576245536813, 1.9225433836557082, NA, NA, NA, -4.596778040074198, -5.607069015469298, NA, NA, NA, -1.0493445378348683, NA, NA, NA, NA, -1.5749722529453734, NA, NA, NA, NA, 0.6523239727088999, 2.0567423069103095, NA, NA, NA, -3.509270508171897, -1.3516965093329671, NA, NA, NA, 3.4100314512733876, NA, NA, NA, NA, 5.661709570086349,

NA, NA, NA, NA, -0.9101472379858135, 2.914764501376232, NA, NA, NA, -1.4955232622785628, 0.5163632710044764, NA, NA, NA, -4.638469129464038, NA, NA, NA, NA, -1.3155096047006314, -1.023906735082899, -2.487899160959333, NA, NA, 3.195054747910441, NA, NA, NA, NA, -0.5169248288562442, NA, NA, NA), .Dim = c(51, 5)),
sd.d = 7.484189469381685
)

list(
d.EX10BID.EX2QW = 1.1637341602057858,
d.EX10BID.Exe5ug = 0.8257901282731981,
d.EX10BID.Insulin = 2.692818787871214,
d.EX10BID.LIR18 = -0.3254700972810159,
d.EX10BID.Met = 2.384005879096936,
d.EX10BID.SU = 3.807596308470061,
d.EX10BID.Sitagliptin = -0.4042698237002247,
d.EX10BID.TZD = 9.347542529388628,
d.EX10BID.placebo = 1.9540034845650103,
d.SU.LIR06 = -2.4871762824322183,
d.SU.LIR09 = 7.006729968492676,
d.SU.LIR12 = -3.8938560393967503,
mu = c(-5.791305488397549, -1.4075301181615507, -1.013926146128699, -1.8954097982385671, -3.560492637242136, -0.9186764313663023, -0.7942889395334382, -2.161873452159309, -2.467688177113445, -2.168457373097732, -2.1119738850458347, -2.7801109696756545, -3.1055071758019954, -3.986212460753312, -3.19778953877456, -2.8376784505515698, -5.653130760146789, -7.467423437283165, -4.823436372126387, -4.2172006377448605, 6.913880168754146, -4.10868964114692, -4.153244911843388, -0.9661476103824551, -1.9830154148591888, -1.47449621813236, -1.7101197685674001, -0.19336992171787248, -2.143080383039722, -1.5391684909333891, -1.7084983402196219, 1.9555698256585878, -0.9824676786051804, -5.694571563871649, 0.8132146103928372, 0.034367149612613224, 0.8163099153822178, -1.3806586283649251, 1.0757939510999337, -3.880915516343329, -2.8503433961314064, -2.902247324233816, -0.21467680887654228, -5.123425055507345, -1.8674972519176452, -3.670138296001716, -7.094129693239151, 2.5393775987911464, 1.27355299993328, -6.993964663172424, -1.998755704348719),
delta = structure(.Data = c(NA, 4.1059867865027195, NA, NA, NA, NA, 1.7778459270512115, 0.4022568905641323, NA, NA, NA, 2.9939585561509876, NA, NA, NA, NA, 6.2582237955380355, NA, NA, NA, NA, 6.307563623363495, NA, NA, NA, NA, 0.42273838670601355, -0.9230464855256786, NA, NA, NA, 2.7519835818601335, NA, NA, NA, NA, -0.9477524757005501, NA, NA, NA, NA, 3.434344367843626, 4.281188906414832, NA, NA, NA, 4.501638123125113, NA, NA, NA, NA, 1.8008500538750651, 0.4141147873936315, 0.53478497151408, NA, NA, -1.2564635202285148, NA, NA, NA, NA, 1.7221926306703124, NA, NA, NA, NA, 5.017830292046046, NA, NA, NA, NA, 5.518299997850061, NA, NA, NA, NA, 0.8804107466545704, 0.10049988408294919, NA, NA, NA, 4.104945940712926, NA, NA, NA, NA, 11.352734529796884, NA, NA, NA, NA, 4.735622183615793, NA, NA, NA, NA, 8.349397208069606, NA, NA, NA, NA, 32.609145997832066, NA, NA, NA, NA,

5.1721902667765125, NA, NA, NA, NA, 6.957507498528236, NA, NA, NA, NA, 1.3423513419770192, NA, NA, NA, NA, 1.4937088354419346, NA, NA, NA, NA, 3.348937415429946, NA, NA, NA, NA, 0.7373976447458276, NA, NA, NA, NA, 1.2016204474964265, NA, NA, NA, NA, 0.8485582629775772, NA, NA, NA, NA, 0.6006277863647217, 1.611724097734392, NA, NA, NA, 1.1497369018043115, 2.0246007861722175, NA, NA, NA, -0.6523336500749328, 0.27936681420073883, NA, NA, NA, 1.0137437631174868, 0.7784655513824699, NA, NA, NA, 5.162625032136913, NA, NA, NA, NA, -0.19718756467884868, 0.23222485150258199, 0.753365682174931, -0.8574516338908733, NA, -1.9083179379528676, -3.675545988647708, -3.9131792171684947, -2.046981240729136, NA, -3.8496570863374076, -3.744481545627679, NA, NA, NA, -0.23514629857518576, 2.7817166543879983, NA, NA, NA, -3.7810249011125925, -0.5731527274012327, NA, NA, NA, -0.30572983690991323, NA, NA, NA, NA, -0.03737953242524772, NA, NA, NA, NA, 0.75116745810341, 1.80784993307773, NA, NA, NA, -0.06097742418473029, -1.1456040964733532, NA, NA, NA, 0.6525919935657694, NA, NA, NA, NA, 6.03963940379872, NA, NA, NA, NA, -1.8229506774258257, 1.893091127640843, NA, NA, NA, -8.665442361775323, -0.45113429403826555, NA, NA, NA, -9.397201329024565, NA, NA, NA, NA, -1.505439325155451, -2.4162078764943153, -3.3402839651745824, NA, NA, 1.8826460990773513, NA, NA, NA, NA, 0.8316166770912887, NA, NA, NA), .Dim = c(51, 5)),
sd.d = 2.021925645136791
)

list(
d.EX10BID.EX2QW = 1.3375870476901888,
d.EX10BID.Exe5ug = -0.20106664910581173,
d.EX10BID.Insulin = 6.480629092593263,
d.EX10BID.LIR18 = 1.0409630874546978,
d.EX10BID.Met = 2.83710364794395,
d.EX10BID.SU = 5.030565458645571,
d.EX10BID.Sitagliptin = 2.5136443289397508,
d.EX10BID.TZD = 5.302844828836797,
d.EX10BID.placebo = 1.3342081052105108,
d.SU.LIR06 = -0.6170229615348719,
d.SU.LIR09 = 2.2763682578371296,
d.SU.LIR12 = -3.7916036121546104,
mu = c(-7.429665694858892, -2.9006139120851873, -1.13844145524712, -2.038451687037297, -2.416041789286858, -1.0587971943713503, -0.9672025403238612, -5.373447115650487, -1.6703300070383857, -2.075737694078856, -1.7423793057395858, -2.6193938281257534, -2.6599089938793883, -1.7520719179129578, -3.884022410303156, -1.6208270194187882, -1.1200594814519147, -8.298105032746736, -5.427397816762944, -4.671135940834692, 8.764588358571094, -4.398469069471638, -2.466059702240992, -1.075516755273885, -0.7369858841258182, -1.5733985294384851, -1.9377393396394778, 1.230740073946463, -2.631301141096952, -0.7363540100137769, -1.10234429793186, -1.4296377793660642, -1.966530690521698, -5.835401666878827, 0.6028237084350838, 0.31744002855388664, 0.9725800332782532, -2.6680294529239315, 2.258095545787132, -3.4969228177537506,

-2.4723484002082095, -2.639395248095126, 1.6918345861816513, -3.256666600258299, -1.9322033960187737, -3.6589847099109516, 3.5620358894856237, 2.2147161751817017, 0.2666717400434485, -5.312735388424251, -2.5920937113254743),
delta = structure(.Data = c(NA, -0.62533017887452, NA, NA, NA, NA, -0.7411233827261985, 0.13688309957660594, NA, NA, NA, 3.9160646407890107, NA, NA, NA, NA, 5.361135501869258, NA, NA, NA, NA, 8.101759950194824, NA, NA, NA, NA, -0.1323382494675046, 1.3716270958759524, NA, NA, NA, 3.6555782901872673, NA, NA, NA, NA, -2.8471217191961906, NA, NA, NA, NA, 3.403981614183505, 4.709716759484515, NA, NA, NA, 4.422570633980912, NA, NA, NA, NA, 0.3642655355381908, -0.03614789628840498, 4.737237634536107, NA, NA, -1.5573511359295527, NA, NA, NA, NA, 4.339876556524576, NA, NA, NA, NA, 5.421440639439291, NA, NA, NA, NA, 4.928216315153906, NA, NA, NA, NA, -1.447417372802176, 3.840093410926224, NA, NA, NA, 3.4434271412144506, NA, NA, NA, NA, 11.86126339192324, NA, NA, NA, NA, 3.973461904684532, NA, NA, NA, NA, -4.782552107542538, NA, NA, NA, NA, 29.768293605543032, NA, NA, NA, NA, 5.12932857033138, NA, NA, NA, NA, 4.077242974990854, NA, NA, NA, NA, 1.063134967126403, NA, NA, NA, NA, -1.1582701846011492, NA, NA, NA, NA, 4.167126090357079, NA, NA, NA, NA, 0.2150883171500242, NA, NA, NA, NA, -3.275823743347682, NA, NA, NA, NA, 1.0863243890604932, NA, NA, NA, NA, 0.4053166822469264, 1.196880067552085, NA, NA, NA, 1.6352306035286528, 1.791201395389736, NA, NA, NA, -5.6225549312700895, 5.189198320028462, NA, NA, NA, -1.1477834714593134, 0.41946393693170064, NA, NA, NA, 2.119084497883178, NA, NA, NA, NA, -0.7462881585185053, -2.3770225906530893, 1.2880962716364606, -0.7685813423602407, NA, -3.538776658784631, -2.548361710339751, -2.6927902634150875, -2.9710154840438814, NA, -1.9137912250311273, -2.810238819026686, NA, NA, NA, 0.17642912367864994, 2.011932113455032, NA, NA, NA, -5.24593658365599, -2.9656126941060195, NA, NA, NA, 1.9392400451204383, NA, NA, NA, NA, 1.5280073677239367, NA, NA, NA, NA, 0.597804169063762, 1.4814432639008572, NA, NA, NA, 1.0710836533001502, -1.620540299671808, NA, NA, NA, 2.6365919372928532, NA, NA, NA, NA, 4.92264274583511, NA, NA, NA, NA, -1.6299898817437555, 1.0871294939763763, NA, NA, NA, 1.2676217340883333, -8.118730539147482, NA, NA, NA, -3.154489448916247, NA, NA, NA, NA, -2.3356097104753624, -2.604732152171071, -3.5261879150227347, NA, NA, 1.8815091031685816, NA, NA, NA, NA, 2.4885225162490814, NA, NA, NA), .Dim = c(51, 5)),
sd.d = 2.5355283399811857
)

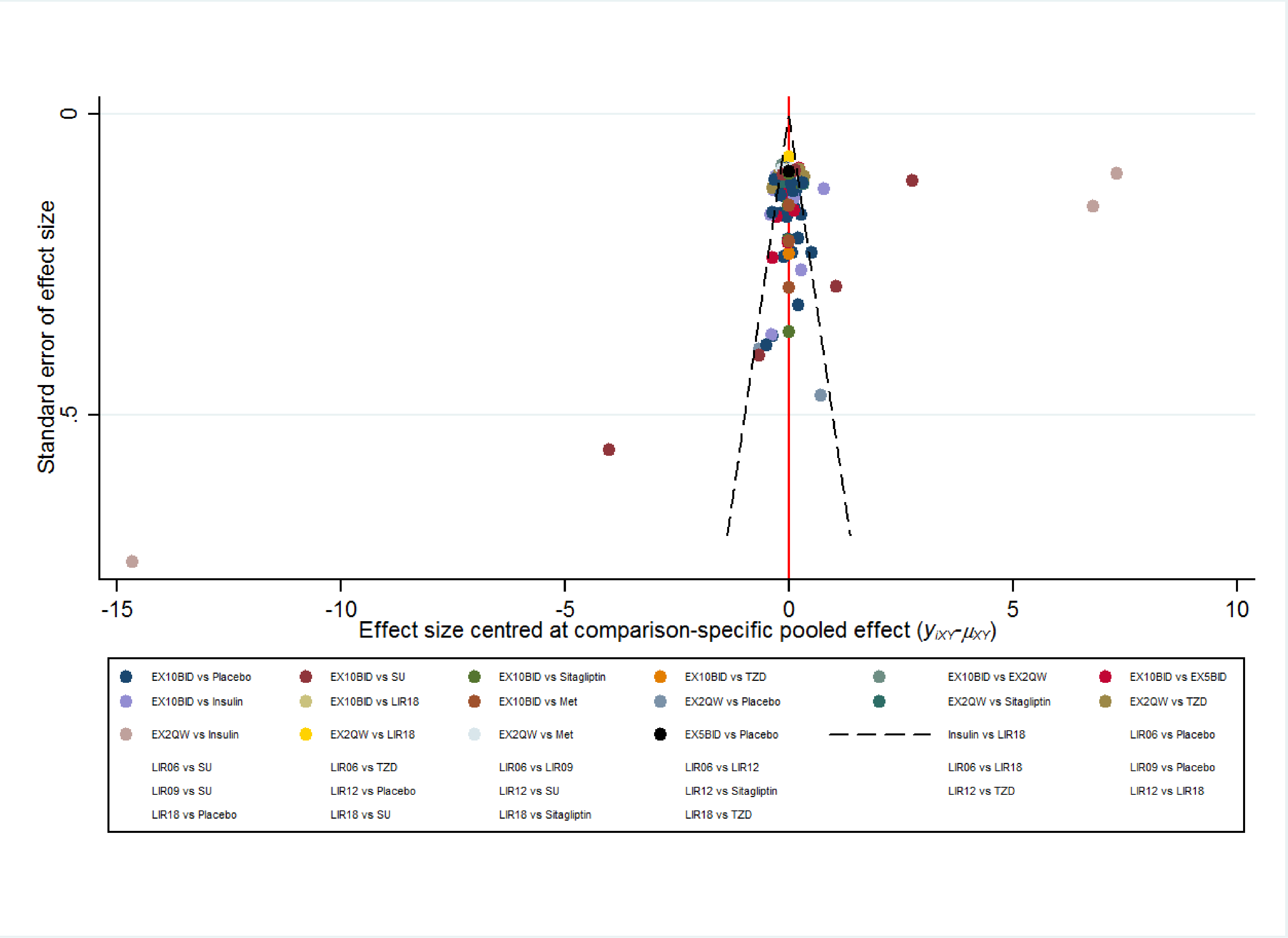

Standard error of effect size
0
.5
-15
-10
-5
0
5
10
Effect size centred at comparison-specific pooled effect ($y_{iXY}-\mu_{XY}$)
EX10BID vs Placebo
EX10BID vs SU
EX10BID vs Sitagliptin
EX10BID vs TZD
EX10BID vs EX2QW
EX10BID vs EX5BID
EX10BID vs Insulin
EX10BID vs LIR18
EX10BID vs Met
EX2QW vs Placebo
EX2QW vs Sitagliptin
EX2QW vs TZD
EX2QW vs Insulin
EX2QW vs LIR18
EX2QW vs Met
EX5BID vs Placebo
Insulin vs LIR18
LIR06 vs Placebo
LIR06 vs SU
LIR06 vs TZD
LIR06 vs LIR09
LIR06 vs LIR12
LIR06 vs LIR18
LIR09 vs Placebo
LIR09 vs SU
LIR12 vs Placebo
LIR12 vs SU
LIR12 vs Sitagliptin
LIR12 vs TZD
LIR12 vs LIR18
LIR18 vs Placebo
LIR18 vs SU
LIR18 vs Sitagliptin
LIR18 vs TZD

**EX10BID**												
-1.14 (-2.34,0.04)	**EX2QW**											
-1.02 (-2.21,0.20)	0.15 (-1.53,1.77)	**Exe5ug**										
**-4.68 (-5.66,-3.68)**	**-3.52 (-4.74,-2.29)**	**-3.67 (-5.18,-2.13)**	**Insulin**									
**-2.64 (-4.38,-0.88)**	-1.48 (-3.46,0.40)	-1.63 (-3.70,0.39)	**2.04 (0.09,3.93)**	**LIR0.6**								
-3.13 (-6.71,0.46)	-1.99 (-5.75,1.77)	-2.10 (-5.88,1.60)	1.55 (-2.13,5.24)	-0.51 (-4.19,3.26)	**LIR0.9**							
**-1.82 (-3.17,-0.44)**	-0.67 (-2.26,0.87)	-0.81 (-2.51,0.95)	**2.86 (1.29,4.40)**	0.82 (-0.95,2.60)	1.30 (-2.42,4.97)	**LIR1.2**						
-0.99 (-2.13,0.13)	0.16 (-1.20,1.49)	0.02 (-1.54,1.55)	**3.69 (2.35,5.00)**	1.65 (-0.04,3.37)	2.14 (-1.50,5.77)	0.83 (-0.41,2.06)	**LIR1.8**					
-1.61 (-3.59,0.39)	-0.45 (-2.59,1.61)	-0.60 (-2.89,1.74)	**3.06 (0.91,5.19)**	1.02 (-1.45,3.57)	1.52 (-2.52,5.48)	0.21 (-2.08,2.45)	-0.62 (-2.75,1.53)	**Met**				
**-6.08 (-7.35,-4.80)**	**-4.94 (-6.54,-3.32)**	**-5.07 (-6.74,-3.36)**	-1.41 (-2.92,0.14)	**-3.44 (-5.26,-1.56)**	-2.95 (-6.52,0.56)	**-4.27 (-5.78,-2.70)**	**-5.11 (-6.47,-3.67)**	**-4.48 (-6.71,-2.17)**	**SU**			
**-2.68 (-4.39,-0.95)**	-1.52 (-3.23,0.15)	-1.67 (-3.69,0.38)	**1.99 (0.15,3.86)**	-0.03 (-2.25,2.18)	0.46 (-3.42,4.33)	-0.86 (-2.65,0.96)	-1.68 (-3.40,0.02)	-1.05 (-3.27,1.17)	**3.40 (1.45,5.38)**	**Sitagliptin**		
**-4.88 (-6.61,-3.19)**	**-3.75 (-5.38,-2.08)**	**-3.89 (-5.91,-1.84)**	-0.22 (-2.00,1.63)	**-2.25 (-4.30,-0.15)**	-1.76 (-5.64,2.06)	**-3.07 (-4.89,-1.27)**	**-3.90 (-5.60,-2.20)**	**-3.28 (-5.45,-1.00)**	1.20 (-0.74,3.13)	**-2.21 (-4.01,-0.41)**	**TZD**	
**-2.07 (-2.93,-1.25)**	-0.93 (-2.30,0.42)	-1.07 (-2.26,0.14)	**2.60 (1.39,3.80)**	0.56 (-1.15,2.28)	1.06 (-2.56,4.68)	-0.25 (-1.62,1.06)	-1.10 (-2.20,0.05)	-0.46 (-2.52,1.61)	**4.01 (2.58,5.32)**	0.60 (-1.16,2.36)	**2.81 (1.09,4.53)**	**Placebo**

Appendix Figure.Networkmeta-analysisresultsof13treatmentsatleast 24w among 37 RCTs.

Treatmentsarereportedinalphabeticalorder.Foruppertriangle,comparisonsbetweentreatmentsshouldbereadfromtoptoleftandtheestimateisinthecellincommonbetweenthe column-definingtreatmentandtherow-definingtreatment.EX5BID:exenatide5μgtwicedaily;EX10BID:exenatide10μgtwicedaily;EX2QW:exenatide2mgonceweekly;LIR0.6:liraglutide0.6 mgoncedaily;LIR0.9:liraglutide0.9mgoncedaily;LIR1.2:liraglutide1.2mgoncedaily;LIR1.8:liraglutide1.8mgoncedaily;SU:sulphanylureas;TZD:thiazolidinedione;MET:metformin.
